# Women’s view on shared decision making and autonomy in childbirth: cohort study of Belgian women

**DOI:** 10.1186/s12884-022-04890-x

**Published:** 2022-07-08

**Authors:** Elke Deherder, Ilse Delbaere, Adriana Macedo, Marianne J. Nieuwenhuijze, Sven Van Laere, Katrien Beeckman

**Affiliations:** 1 VIVES University of Applied Sciences, Doorniksesteenweg 145, 8500 Kortrijk, Belgium; 2 Student master management and policy of health care, department of Public Health and Nursing and Midwifery Unit, Vrije Universiteit Brussel, UZ Brussel, Brussels, Belgium; 3 Research Centre for Midwifery Science Maastricht, Zuyd University / CAPHRI, Maastricht University, Universiteitssingel 60, 6229 ER Maastricht, the Netherlands; 4 Vrije Universiteit Brussel, Interfaculty Center Data processing & Statistics, Laarbeeklaan, 103 Brussels, Belgium; 5 Vrije Universiteit Brussel, Universitair ziekenhuis Brussel (UZ Brussel), Faculty of Medicine and Pharmacy, Public Health, Nursing and Midwifery Research Unit, Laarbeeklaan 101, 1090 Brussels, Belgium; 6 Verpleeg- en vroedkunde, Centre for Research and Innovation in Care, Midwifery Research Education and Policymaking (MIDREP), Universiteit Antwerpen, Antwerpen, Belgium

**Keywords:** shared decision making, pregnancy, childbirth, autonomy

## Abstract

**Background:**

Health care providers have an important role to share evidence based information and empower patients to make informed choices. Previous studies indicate that shared decision making in pregnancy and childbirth may have an important impact on a woman’s birth experience. In Flemish social media, a large number of women expressed their concern about their birth experience, where they felt loss of control and limited possibilities to make their own choices. The aim of this study is to explore autonomy and shared decision making in the Flemish population.

**Methods:**

This is a cross-sectional, non-interventional study to explore the birth experience of Flemish women. A self-assembled questionnaire was used to collect data, including the Pregnancy and Childbirth Questionnaire (PCQ), the Labor Agentry Scale (LAS), the Mothers Autonomy Decision Making Scale (MADM), the 9-item Shared Decision Making Questionnaire (SDM–Q9) and four questions on preparation for childbirth. Women who gave birth two to 12 months ago were recruited by means of social media in the Flemish area (Northern part of Belgium).

Linear mixed-effect modelling with backwards variable selection was applied to examine relations with autonomy in decision making.

**Results:**

In total, 1029 mothers participated in this study of which 617 filled out the survey completely. In general, mothers experienced moderate autonomy in decision-making, both with an obstetrician and with a midwife with an average on the MADM score of respectively 18.5 (± 7.2) and 29.4 (±10.4) out of 42. The linear mixed-effects model showed a relationship between autonomy in decision-making (MADM) for the type of healthcare provider (*p* < 0.001), the level of self-control during labour and birth (LAS) (*p* = 0.003), the level of perceived quality of care (PCQ) (*p* < 0.001), having epidural analgesia during childbirth (*p* = 0.026) and feeling to have received sufficient information about the normal course of childbirth (*p* < 0.001).

**Conclusions:**

Childbearing women in Flanders experience moderate levels of autonomy in decision- making with their health care providers, where lower autonomy was observed for obstetricians compared to midwives. Future research should focus more on why differences occur between obstetrics and midwives in terms of autonomy and shared decision-making as perceived by the mother.

## Background

The ethical need to respect autonomy of patients is recognized as well as the desire of patients to be involved in the decision-making process regarding their medical condition or treatment [1]. It is usually conceptualized as ‘patient centeredness’, which is a broad and variably interpreted concept [1]. Each healthcare discipline seeks a way to imply decision making.

Within the NICE (National Institute for Health and Care excellence) guidelines and WHO (World Health Organization) intrapartum care for healthy women and babies, shared decision-making is defined as a woman’s right to be involved in making choices about her care [2, 3]. Women value the opportunity to fully participate in the care planning intended for them, including the ability to understand and apply the best available evidence to their individual situations [4, 5]. The involvement of women in pregnancy and during childbirth in the decision making process has a profound effect on the birth experience and on satisfaction with care [6, 7]. Previous studies indicated that pregnant women value the support of involved caregivers, but they also want to participate in decision making during the pregnancy and birth process [6].

Christiaens & Bracke [8] and Larkin et al. [9] showed that there are four elements determining the level of satisfaction in childbirth. First, meeting preset expectations is the most important determining factor for a satisfying birth experience (feeling in control). Next to that, also personal control and pain perception are important determinants and finally, self-assured women showed greater satisfaction with their birth, particularly when they experienced adequate support from the midwife and physician [8–10]. It is the belief that when the woman can make her own choice it reinforces the sense of control, and assigning decision responsibility to the woman may be part of this [7, 9].

Birth satisfaction, in turn, has an impact on the mother’s health and relationship with her child. A traumatic experience and lack of fulfillment with the course of childbirth can cause postpartum depression and post-traumatic stress syndrome. As such, women relive their childbirth in nightmares and flashbacks. Traumatic experiences and dissatisfaction negatively impacts the success and progression of breastfeeding and mother-child bonding [10].

Osamor & Grady [11] and Anderson & Eswaran [12] showed that greater autonomy in decision making conversations is essential for better maternal and child health outcomes. It ensures greater child survival and higher levels of well-being  [11, 12].

Shared Decision Making (SDM) is a practice that integrates autonomy and involvement in decision-making. SDM is defined as: “an approach in which health care providers and health care consumers share the best available evidence, encouraging health care consumers to consider options and make informed choices” [6, 13]. SDM is based on the acceptance that self-determination is an achievable goal and that healthcare providers should support patients to achieve this goal (whenever possible). However, self-determination does not mean leaving patients to their fate. SDM recognizes the need for support and will pursue good caregiver-care receiver relationships, respect for each other’s knowledge, competencies, and experiences [6, 13].

Applying SDM in the perinatal period is challenging, particularly during labour and childbirth. Research on SDM in the perinatal period is rare, especially for SDM in the dynamic process of labour and birth [6, 14]. Sometimes urgent (on the spot), life-saving decisions have to be made [6, 14]. Nevertheless, clear and open communication with patients is always necessary, as is the assurance that all information has been properly understood [6, 14]. Listening to the patient’s point of view, properly motivating the medical decisions that have to be made and explicitly asking for approval are examples good SDM practice [6].

The benefits of SDM for the patient include improved knowledge, reduced anxiety, increased self-confidence, and increased awareness of risks [1, 15]. There are also disadvantages associated with SDM: it is more time-consuming, it may be more challenging for certain healthcare disciplines (labor and birth) and not every healthcare provider is properly trained in SDM [6, 13–15]. Moreover, not every patient desires to be involved in decision making in the same way. Nieuwenhuijze, et al. [16] emphasized that the degree to which women want to participate in decision making regarding their care might vary [15, 16]. Women’s involvement also seems to surface from feeling that they could challenge decisions made by others if the need arises instead of making decisions themselves [16]. It is important to communicate about SDM at the start of the health care interaction.

A few years ago #metoo, #genoeggezwegen (in English: #enoughsilence) appeared in the Flemish social media. With this hashtag, women expressed their negative and often traumatic birth experiences in blogs, opinion articles, Facebook posts and other channels. The common threat throughout their complaint was loss of control, experiencing very limited opportunities to express their own choices or experiencing ignoration of expressed choices.

As a result of #genoeggezwegen, we wanted to explore the extent to which involvement in midwifery and obstetric care contributes to the childbirth experience. The aim of this study was to explore Flemish women’s experience with labour and birth, satisfaction with care received, and to find out to what extent the Flemish woman has been involved in this care and the decision making with regard to this care. To our knowledge, this is the first Belgian study on this topic.

## Methods

### Design and setting of the study

This is a non-interventional cross-sectional study on the birth experience of Flemish women. The study took place in the northern region of Belgium, i.e. Flanders.

### Instrument

From the current literature [7–9], it became apparent that birth experience is compiled by several different aspects: support by the healthcare provider and partner, relationship with the healthcare provider, experience of pain, self-control and experienced control in decision making. In order to measure these different aspects, we used a combination of several validated scales leading to a self-assembled instrument.

First, the Mothers Autonomy Decision Making (MADM) scale [17] was used to explore women’s experiences of decision making. Secondly, the Pregnancy and Childbirth Questionnaire (PCQ) [18] on the quality of care during pregnancy and childbirth was incorporated. Next, the Labour Agentry Scale (LAS) was questioned to assess self-control during labour and birth. As a fourth scale, the 9-item Shared Decision Making Questionnaire (SDM-Q-9) [19, 20] was used to measure the degree of shared decision-making on the topic of pain management. Lastly, also information on the preparation for birth (four questions), birth experience (one question) and personal and obstetric information was asked.

A draft questionnaire was presented to a sample of the target population (women who gave birth 2 to 12 months ago) at two different times. During the two focus group discussions, the “thinking aloud” method was used. Seven women participated in the focus groups. The participants mainly had suggestions related to language (unclear words or professional jargon), additional explanations to certain items of the questionnaire (e.g., additional answer options), adjusting the order of the different sections of the questionnaire and converting complex answer options to a slider. The participants suggested adding a new response option (neutral) at the MADM and SDM-Q9 scale to avoid skipping questions and thereby avoiding drop out.

### Mothers Autonomy Decision Making (MADM)

This scale focuses on the mother’s autonomy in decision making and thus will be used as a central measure throughout this research. In the original version of the MADM, the aim is to identify one healthcare provider, specifically general practitioner, midwife or obstetrician, with whom the patient had decision-making discussions, and complete the questionnaire accordingly. In Belgium, typically a midwife and an obstetrician are involved during labour and birth. As a result, the choice was made to offer the MADM scale twice in this study. The participant completes the MADM once for conversations held with the midwife and once for conversations held with the obstetrician. The MADM scale was initially created with six response options for the seven items included. This Likert scale ranged from “totally disagree” (one point) to “totally agree” (six points). Participants from the two focus groups suggested to add a new response option (neutral) which was awarded 3.5 points. Participants stated that not providing this option could lead to loss of data and inability to calculate the total score of autonomy. The sum score was later on calculated with a minimum score of 7 and a maximum score of 42. A higher score indicates a greater experience of autonomy in decision-making.

### Pregnancy and Childbirth Questionnaire (PCQ)

This scale focuses on quality of care during pregnancy and childbirth. We only used the birth- subscale (7 generic items) and 4 of the 5 items of the collaboration scale. We included the following questions: ‘I felt that the caregivers were close enough to me throughout the delivery’; ‘I was well received at the delivery room’ and ‘I felt left to my own devices at the delivery room’. We didn’t included the item ‘referral to hospital providers during my delivery went well’, since this item is not applicable in our national care setting where 99% of women attend labour and birth in a hospital setting.. Questions were formed in positive and negative statements, rated on a five- point Likert scale from 1 (totally agree) to 5 (totally disagree). After recoding, higher scores indicated higher quality of care. For this scale, the minimum score is 10 and the maximum score is 50.

### Labour Agentry Scale (LAS)

This scale focuses on self-control during labour and birth. The original LAS with 29 items was developed by Hodnett and Simmons- Tropea (1987). The shorter version of the LAS consists of 10 questions [16]. The short version was chosen primarily to reduce the length of the total questionnaire. The Dutch version of the short LAS was used in the Netherlands by Nieuwenhuijze, M., et al. [16]. After translation, 11 items were retained as suggested by the focus group, since the item ‘I felt helpless (powerless)’ was translated into two separate items. The reason for this is based on the difference in meaning of ‘powerless’ and ‘helpless’ in the Dutch language. These 11 items consist of 6 positively formulated statements and 5 negatively formulated statements. The positive items are reversed for analysis and summated to a total score. For this scale, the minimum score is 11 and the maximum score is 77. Higher scores indicate a higher sense of control.

### The 9-item Shared Decision Making Questionnaire (SDM-Q- 9)

This scale was created as a brief patient-report instrument for measuring Shared Decision Making in clinical encounters. Normally, the participant fills out the topic of the conversation with the caregiver. We chose to define the topic ourselves, namely pain management, to obtain data can be compared more easily. The scale consists of nine questions with six response options ranging from “totally disagree” (score 0) to “totally agree” (score 5). Analogue with the MADM scale, the focus group to evaluate the instrument preferred to include a seventh response option (neutral). Similarly, this additional option was given a value of 2.5 points. The aggregated scores over all items of the SDM-Q-9 lead to a total raw score between 0 and 45, with 0 indicating the lowest and 45 indicating the highest level of perceived SDM.

### Other questions in the self-assembled instrument

Information on the preparation for labour and birth (four questions) and personal and obstetric information were asked in addition to the (sub)scales. The composition of the personal characteristics and the obstetric characteristics was based on previous research on birth experience and on the different scales used.

The four questions about preparing for childbirth were developed by the researchers themselves. The four questions were: ‘I felt sufficiently prepared for the birth’; ‘The given information fulfilled my needs’; ‘The quality of the provided information was good’ and ‘I have received sufficient information about the normal course of childbirth’.

The personal characteristics were: age (at the time of birth), education and place of birth. The obstetric characteristics were: parity, initiation of labour started, gestational age, the birth, use of epidural anesthesia, presence of problems with mother and/or baby during labour, presence of problems with mother and/or baby during birth and presence of problems with mother and/or baby immediately after birth. The questions about presence of problems (or complication) were self-completing. The following question was asked: ‘Were there any problems with you and/or your baby during labour? If so, what was the problem?’ This question was repeated twice more for the period of birth and for the period after birth.

### Data collection, study population and ethical concerns

Data were collected between 3 March 2020 and 15 June 2020 by means of an anonymous, structured, online questionnaire (LimeSurvey®), distributed via Facebook, Instagram and LinkedIn. Participation in this study was voluntary and anonymous. Ethics committee approval was obtained on 4 November 2019 (AZGS2019058) and participants gave informed consent before participation.

The study population consists of all postpartum women who gave birth between two and 12 months before participation in Flanders. Inclusion criteria were: having sufficient knowledge of the Dutch language, being 18 years or older, having given birth at least once, being in the postpartum period (between two and 12 months postpartum), having given birth in Flanders and voluntarily agree to participate in this study. The call was launched on March 3rd 2020, and a reminder sent April 23rd and May 11th 2020.

### Data analysis

Descriptive statistics were reported in means and standard deviations for continuous variables and in absolute and relative frequencies for discrete variables. For the different scales, we report median, percentiles, interquartile range (IQR) and range.

Analysing the relations with scores on the MADM- scale, a linear mixed-effects model was build using backwards model selection based on the Akaike Information Criterion (AIC). In that modelling, initially all other variables were entered to test for association. In the final model, only statistically significant covariables were retained for reporting. Final estimation was obtained using restricted maximum likelihood (REML). For reporting, beta estimates, standard deviations, t- and *p*- values were reported. A *p*-value of 0.05 was considered as statistically significant. Data were analysed with IBM® SPSS® (version 25).

## Results

### Sample

A total of 1029 participants responded and 617 participants completed the questionnaire. Because of online sampling, it is not possible to calculate the sampling fraction and sampling response.

### Patient characteristics, obstetrical characteristics, complications and preparation to childbirth

Half of the women who participated in this study were 18 to 29 years old (49.8%). The majority of participants has completed a Bachelor or Master degree (83.1%) and approximately half of participating women were primiparous (54.3%). In 61.1% of the respondents, labour started spontaneously and almost 50% of women gave birth naturally. The majority gave birth at term and reported no problems during labour (71.2%), at the time of birth (72.8%) or after birth (80.4%).

In addition, when analysing the experience of preparation for childbirth, 80.2% of the participants felt adequately prepared for their birth and the same percentage of women (80.2%) indicated that the received information fulfilled their needs. Most women (82.2%) were satisfied with the quality of the received information and felt they received sufficient information about the normal course of child birth (78.9%) These results are depicted in Table 1.

**Table 1:** Descriptive statistics of sample recruited

Variable	N	(%)
Patient characteristics		
Age at giving birth		
≥ 18 to 29 years	307	(49.8)
≥ 30 to 34 years	249	(40.4)
≥ 35 years	61	(9.9)
Education		
No education, primary education or secondary education	104	(16.9)
Bachelor degree	283	(45.9)
Master degree	230	(37.3)
Parity		
1	335	(54.3)
2	216	(35.0)
≥ 3	66	(10.7)
Obstetrical characteristics		
Length of gestation		
≤ 37 weeks	75	(12.2)
≥ 38–40 weeks	333	(54.0)
> 40 weeks	209	(33.9)
Mode of birth		
Natural birth	298	(48.3)
Vacuum extraction	29	(4.7)
Primary c-section	237	(38.4)
Secondary c-section	53	(8.6)
Epidural analgesia		
Yes	377	(61.1)
No	240	(38.9)
Complications		
Complications during labour (woman and/or baby)		
Yes	177	(28.7)
No	439	(71.2)
Complications during birth (woman and/or baby)		
Yes	168	(27.2)
No	449	(72.8)
Complications after birth (woman and/or baby)		
Yes	121	(19.6)
No	496	(80.4)

Table 2 presents the results on how prepared women felt to give birth, indicating a preparedness of about 80%.

**Table 2:** Descriptive statistics of preparation for childbirth

Preparation for childbirth		
‘I felt sufficiently prepared for the birth’		
Agree	495	(80.2)
Neutral	66	(10.7)
Don’t Agree	56	(9.1)
‘The given information fulfilled my needs’		
Agree	495	(80.2)
Neutral	69	(11.2)
Don’t Agree	53	(8.6)
‘The quality of the provided information was good’		
Agree	507	(82.2)
Neutral	75	(12.2)
Don’t Agree	35	(5.7)
‘I have received sufficient information about the normal course of childbirth’		
Agree	487	(78.9)
Neutral	77	(12.5)
Don’t Agree	53	(8.6)

A large proportion of primiparous women responded to our questionnaire: more than half of our responders gave birth for the first time (54.3%), whereas primiparous women make up 45% of the general Flemish population giving birth. Nonetheless, age categories of the respondents are similar to the ones in the general population (49.8% of respondents aged 29 or younger versus 44% in the general population; 40.4% of respondents aged 30–34 years versus 37.2% in the general population), only the group of women aged 35 or older is underrepresented in our study population (9.9% versus 18.6%) [21]. Natural births are underrepresented in our sample size; in the general population, 69.4% of women deliver vaginally (48.3% in this sample size) and 21% deliver by caesarean section; 11% primary section and 9.5% secondary section (respectively 47, 38.4 and 8.6% in this sample size). Births by vacuum extraction are underrepresented in this sample size (4.7% versus 9.7% in the general population).

### Results of subscales

Subsequent the mean score in the different subscales were calculated (Table 3).

**Table 3:** Scores obtained for the different subscales questioned in the questionnaire

	Mean score	±	SD	Median	P_25_-P_75_	(IQR)	Min	–	Max
MADM midwife	29.4	±	10.4	30	22–39.5	(17.5)	7	–	42
MADM obstetrician	18.4	±	7.2	19.5	13–22.5	(9.5)	7	–	42
SDM-Q9	27.5	±	12.3	27.5	27.5–37.5	(10)	0	–	45
Birth experience	7.1	±	2.6	9	9–9	(0)	1	–	9
PCQ	37.9	±	6.0	38	34–43.	(9)	13	–	50
LAS	57.3	±	12.4	59	50–66.5	(16.5)	11	–	77

*MADM* Mother’s Autonomy in Decision Making, *SDM-Q9* 9-Item Shared Decision Making Questionnaire, *PCQ* Pregnancy and Childbirth Questionnaire, *LAS* Labor Agentry Scale, *SD* Standard deviation, *P*_*25*_ 25th percentile, *P*_*75*_ 75th percentile, *IQR* Interquartile range, *Min* Minimum, *Max* Maximum

The perceived autonomy of women in decision-making conversations (MADM) with midwives was 29.4 (±10.4), whereas in decision-making conversations with obstetricians, the MADM-score was 18.38 (±7.2) on a total score of 42 (*p* < 0.001). Furthermore, the perceived shared decision-making in pain management (SDM–Q9) has a mean of 27.5 (±12.3) out of a total of 45. Finally, more than 75% of the participants reported a very positive birth experience with a score of 9 out of 9; the mean score was 7.1 (±2.6).

The Pregnancy and Childbirth Questionnaire (PCQ) is a measure for the quality of care received. The average score in our sample size was 37.9 (±6.0) out of 50 which indicates a rather high satisfaction with the quality of care. Also the perceived self-control during labour and birth has an average of (57.3 ± 12.4), as measured by the Labor Agentry Scale (LAS).

#### Relationships with autonomy in decision making of the mother in the relation with midwives and obstetricians

In Table 4, the Mother’s Autonomy in Decision Making (MADM) scores are summarized for the scores given by the mother. When observing the descriptive variables, we see a difference in the autonomy in decision making, where the MADM score for the midwife scored higher compared to the obstetrician. These differences were observed over all patient characteristics (age, education level and parity of the mother), over all obstetrics characteristics (length of gestation, mode of birth and having received epidural analgesia or not), over the perceived complications (during labour, during birth and after birth), and over all levels of preparedness (questioned subjectively).

**Table 4:** Descriptive statistics of obtained by the Mother’s Autonomy in Decision Making (MADM) scale for both scoring the relation with the midwife and the obstetrician

Variable	Mean ± SD			
	MADM Score for midwife		MADM Score for obstetrician	
Patient characteristics				
Age at giving birth				
≥ 18 to 29 years	29.0	± 10.3	17.9 ±	6.8
≥ 30 to 34 years	29.7	± 10.6	18.8 ±	7.7
≥ 35 years	30.1	± 9.9	20.2 ±	6.9
Education				
No education, primary education or secondary education	31.4	± 9.9	17.4 ±	6.0
Bachelor degree	29.9	± 10.2	17.7 ±	7.0
Master degree	27.8	± 10.6	19.9 ±	7.7
Parity				
1	28.8	± 10.6	18.1 ±	7.4
2	29.6	± 10.1	19.0 ±	7.1
≥ 3	31.2	± 9.7	19.0 ±	6.6
Obstetrical characteristics				
Length of gestation				
≤ 37 weeks	28.4	± 11.1	18.5 ±	6.3
≥ 38–40 weeks	28.7	± 10.1	18.5 ±	7.3
> 40 weeks	30.8	± 10.3	18.4 ±	7.4
Mode of birth				
Naturally	29.7	± 10.8	18.2 ±	7.1
Vacuum	28.4	± 9.0	21.3 ±	6.0
Primary c-section	29.5	± 9.8	17.9 ±	7.3
Secondary c-section	27.2	± 11.1	20.6 ±	7.4
Epidural analgesia				
Yes	28.9	± 10.0	19.2 ±	7.2
No	30.1	± 10.8	17.3 ±	7.1
Complications				
Complications during labour (woman and/or baby)				
Yes	26.6	± 10.9	17.6 ±	7.4
No	30.5	± 9.9	18.8 ±	7.1
Complications during birth (woman and/or baby)				
Yes	27.9	± 10.4	17.6 ±	7.4
No	30.0	± 10.3	18.8 ±	7.1
Complications after birth (woman and/or baby)				
Yes	26.9	± 10.1	17.5 ±	7.2
No	30.0	± 10.4	18.7 ±	7.2
Preparation to child birth				
‘I felt sufficiently prepared for the birth’				
Agree	30.4	± 10.1	18.8 ±	6.9
Neutral	27.7	± 9.9	17.5 ±	7.5
Don’t Agree	22.4	± 10.5	16.4 ±	8.7
‘The given information fulfilled my needs’				
Agree	30.5	± 10.0	18.9 ±	6.9
Neutral	27.3	± 10.0	17.0 ±	7.2
Don’t Agree	21.6	± 10.8	16.1 ±	8.8
‘The quality of the provided information was good’				
Agree	30.8	± 9.7	18.9 ±	6.8
Neutral	24.0	± 10.4	16.0 ±	7.6
Don’t Agree	20.5	± 11.2	17.2 ±	10.3
‘I have received sufficient information about the normal course of childbirth’				
Agree	30.9	± 9.8	19.2 ±	7.0
Neutral	25.2	± 10.0	16.5 ±	6.5
Don’t Agree	21.0	± 10.2	14.9 ±	8.1

*SD *standard deviation

Statistical inference between different characteristics and the MADM scores obtained by midwives and obstetricians occurred by means of a linear mixed-effects model, since every mother scored both the midwife and the obstetrician. A random intercept per mother was entered into the model to include the level of clustering (pairedness) between these two measures. Estimates of the characteristics in relation with the MADM score can be found in Table 5.

**Table 5:** Linear mixed-effects model estimates for the MADM scores after backwards model selection

Variable	Β estimate	±	SD	T value	*P* value
Healtcare provider					< 0.001
Obstetrician	*Ref.*				
Midwife	10.889	±	0.452	24.082	< 0.001
LAS	0.068	±	0.023	2.934	0.003
PCQ	0.490	±	0.047	10.352	< 0.001
Epidural analgesia					0.026
No	*Ref.*				
Yes	1.114	±	0.500	2.227	0.026
‘I have received sufficient information about the normal course of childbirth’					< 0.001
Agree	*Ref.*				
Neutral	−1.504	±	0.724	−2.077	0.038
Don’t agree	−4.070	±	0.863	−4.716	< 0.001

*LAS* Labour Agentry Scale*, PCQ* Pregnancy and Childbirth Questionnaire

Here we observed that on average the MADM score for the midwife is 10.889 (± 0.452) higher than that for the obstetrician (*p* < 0.001). When the perceived self-control during labour and birth, measured by the LAS scale, was higher also the MADM scored higher (*p* = 0.003). When the LAS subscale scored one unit higher, the MADM score increased on average by 0.068 (± 0.023). When the perceived quality of care received, measured by the PCQ scale, was higher also the MADM scored higher (*p* < 0.001). When the PCQ subscale scored one unit higher, the MADM score increased on average by 0.490 (± 0.047). Receiving epidural analgesia during childbirth resulted in an average increase of 1.114 (± 0.500) compared to no epidural analgesia (*p* = 0.026). If the mother indicated to be better informed about the normal course of childbirth, the average MADM score was higher (*p* < 0.001). When comparing the neutral answer to that question to the category that agreed with the statement, an average decrease of 1.504 (±0.724) in MADM was observed (*p* < 0.038). Comparing the mothers who did not agree to the mothers who do agree, we observed that the mothers who did not agree have a decrease of on average 4.070 (± 0.863) on the MADM scale.

## Discussion

Flemish women are in general satisfied with the received care during childbirth. Some aspects of care during labour and birth, such as sufficient preparation and information of good quality, have an important impact on the level of autonomy in decision making. Next to that, a couple of characteristics related to the course of labour and birth (e.g. epidural analgesia, mode of birth, complications) contribute to the perceived autonomy as well.

### The experience of autonomy in decision making

This study shows that women experience higher scores of autonomy when participating in decision-making conversations with a midwife. This result was also found in earlier studies [17, 22]. When analysing the degree of autonomy according to the type of health care provider, it can be concluded that the participants of this study experienced lower autonomy (median = 30.85) when participating in decision-making conversations with a midwife compared to the participants from similar studies in Canada (median > 40) or the Netherlands (median = 35) [17, 22]. Current differences in models of care, health professional education, regulatory standards, and compensations for prenatal visits likely affect the time available for SDM and emphasis placed on the SDM process [17]. It can explain the difference in results between Belgium, the Netherlands and Canada.

This also applies to autonomy with respect to decision making conversations with an obstetrician. Respondents in this study reported a score of autonomy (median = 19.5) that was lower than those in the study by Vedam et al. [17] (median > 29) and Feijen-de Jong et al. [22] (median = 31). In this study as well as in previous studies [17, 22], these differences in the degree of autonomy with respect to the type of caregiver were significant.

Since we did not find other studies on this topic, additional research is needed to be able to get more general statements about the degree of autonomy women experience worldwide during perinatal care.

In addition, the difference in the degree of autonomy with respect to the type of care provider confirms the need for specific research including determinants that could help explain these differences. One determinant might be the provided time for a consultation. Which is short in case of a visit with an obstetrician, resulting in handling medical aspects in particular, while midwives try to spend more time on the preparation of birth and on shared decision-making. The efforts of midwives in shared decision-making and “women-centred” care could be an explanatory factor for the findings in our study [17]. Further research is necessary as it is not the intention of this study to compare the degree of autonomy women experience between different health care providers.

### Important factors in the experience of autonomy in decision-making

Women who perceived low self- control (LAS) reported significantly lower autonomy scores. Nieuwenhuijze et al. [16] established that a positive experience contributes to women’s sense of accomplishment, self- esteem, feelings of competence and well- being. Women with a high degree of self- control, are more likely to feel able to participate in decision making. Furthermore, pain and anxiety during labour may affect the autonomy in decision making [23]. This may also explain why women, who gave birth with epidural analgesia in our study, experienced higher scores of autonomy when participating in decision making with an obstetrician compared to participants who did not receive analgesia. In Belgium a high number of women choose for EDA [21].

Another factor when women may feel “loss of control” is the case when quick decisions have to be made [24]. Our study also found lower MADM – scores in interaction with the care providers when complications occurred during childbirth and in the postpartum period. The lower MADM-scores in interaction with the midwife may be explained, because women count on the midwife to explain the actions that are taken in the event of complications, while they expect this to a lesser extent from the obstetrician as he/she is busy taking care of complications. We need to pay attention to possible traumatic events. Literature shows that women who underwent “traumatic” experiences felt less autonomous when participating in decision-making [25, 26]. Therefore it is important to address possible complications in preparatory sessions as well as after birth the reason behind and actions taken should be discussed. Understanding what happened reduces the chance of post-traumatic stress [27]. As the ‘complications’ in this study were self-reported, the perception can have been subjective. Feelings of stress and additional actions to cope with the complications may feed the perception of less autonomy in decision making.

Preparation for childbirth, particularly the quality of the information received, appears to have an important influence on the extent to which women experience autonomy in decision making as well. This was also the case for women who received sufficient information about the normal course of childbirth. As described by Tully, & Ball [24] care providers should pay attention that the provided information should not only be based on scientific data (“evidence based”) but also communicated in a simple, comprehensive manner [24]. These sessions/moments can also serve to educate women about their right in autonomy and respect for their choices and preferences, which can help avoid disrespectful perinatal concerns [28].

Furthermore both midwives and obstetricians should pay attention to the process of shared decision-making and show respect for the decisions women make. The integration of shared decision making in perinatal care has shown to have a positive effect on the birth experience and on satisfaction with the care provided [6]. The introduction of a birth plan and dialogue between mother and health care provider in the prenatal phase could be an important action in this matter. A birth plan is a written plan in which women express their wishes and preferences for labour, birth and the post-partum. Studies show that women who have a birth plan felt more involved in decision-making and felt more respected [29–31]. Elwyn, G., et al. [13] indicates that SDM consists of three steps (choice talk, option talk and decision talk). Both preparing for childbirth using extensive information (choice and option talk) and preparing a birth plan (decision talk) contribute to the application of SDM in midwifery care [13, 29].

A last element found in our study is the result of higher perceived quality of care (PCQ) in relation to, women experiencing more autonomy. Women experience a positive sense of self from receiving positive affirmations, effective communication with care- givers and experiencing mutually trusting relationships [32]. The results of this study may be useful at the micro level in formulating or modifying local protocols and guidelines promoting respectful perinatal care.

Findings from international literature also reveal that highly medicalized models of care and some features of midwifery care (performing interventions without providing clear indications or explanations to the women receiving them) can diminish women’s sense of self [32]. Investment in a more integrated, centralized childbirth care may also require adjustment of the financing system, in that healthcare providers should be paid in a different way and not merely by performance. This could ensure that healthcare providers feel less pressure and have more time available to pay attention to the decision-making process.

The training of doctors (obstetricians) and midwives can also be an important “tool” for acquiring knowledge and skills about respectful maternity care, women’s autonomy and shared decision-making. The integration of SDM in daily practice demands for modification of communication practices of health care providers [33].

### Strengths and weaknesses

To the best of our knowledge, this is the first study on this topic in Flanders and in Belgium, which makes future comparison possible.

The findings show that the degree to which women experience autonomy when participating in decision-making discussion with health care providers is not higher in Flanders than in other high-income countries, on the contrary it seems lower. This confirms that autonomy, in particular the respect for women’s choices and preferences, is not yet fully respected in Flemish perinatal care.

The focus on two main care providers, midwife and obstetrician, during childbirth is what makes this study unique. Examining factors not previously analyzed in relation with autonomy in decision making, especially in a fairly recent topic in scientific literature such as quality of care; sense of control; quality of information, … are an important contribution of this study.

A limitation of this study is the mode of sampling, recruitment by social media, and as such, this study is subject to selection bias. Long questionnaires can also lead to lower response rates. Furthermore, as the recruitment channels used were mainly social networks such as Facebook, Instagram and LinkedIn, women with no access to the internet of women who have no social media accounts were excluded from this study. However, the number of participants are comparable to other studies in the field and also in studies where women are directly invited often certain groups (e.g. women with lower level of education) decline participation.

One in three women in our study had a cesarean section; as this mode of birth has been discussed with the woman during pregnancy, the large proportion of women with a cesarean section may bias the results related to the MADM-scale in a positive way. As indicated in the results, certain personal and obstetric characteristics of the study population are underrepresented compared to the general Flemish population. This can be attributed to the specific way of recruitment.

The questionnaire referred to experiences during labour and birth. It is not clear to what extent experiences during pregnancy impacted the answers of participating women.

The questionnaire was constructed based on the current organization of obstetric care in Belgium. Labour and birth usually take place in the presence of one midwife and one obstetrician in a hospital setting. Women who gave birth at home (0.7% of deliveries) were excluded from the study.

Another limitation of the study is the adaptation of the MADM and SDM-Q9 with the inclusion of an additional ‘neutral’ response category. Reliability and validity could therefore not be fully guaranteed. However, we do believe the impact of this change will be marginal, since the value assigned to that item was the mean of the preceding and succeeding item, resulting in a same minimum and maximum for both scales.

We chose to work with validated existing scales. The current, known limitations of these scales remain.

Part of our study took place during the COVID-19 pandemic. In Belgium the COVID-19 pandemic started in the second half of March 2020, which was the final month of data collection. As such, only a limited number of women (32 of the 617) gave birth in this period. To be able to find any differences between the period before and during the COVID-19 pandemic would lack from statistical power. Therefore, we decided to leave this variable out of the analysis. Nevertheless, it was observed that other studies indicated an effect of the COVID- 19 pandemic on the perception of quality of care and the degree of autonomy in shared decision making [34, 35].

## Conclusion

According to our study, women in Flanders mainly experience moderate autonomy in decision-making, both with an obstetrician and a midwife. Next to the type of healthcare provider, MADM scores were related to the level of self-control during labour and birth, the level of perceived quality of care, having epidural analgesia during childbirth and feeling to have received sufficient information about the normal course of childbirth. At a time of increasing demand for patient involvement, it is undeniable that more attention needs to be paid to autonomy and shared decision-making in Belgium. Caregivers should be informed about the importance of SDM and trained to apply SDM in daily maternity care. Care receivers should be informed about the possibility of SDM and also motivated to act on it.

Future research should focus more on explaining factors of perceived autonomy and shared decision-making of the mothers and the evaluation on how to include shared decision-making into current practice.

## Data Availability

The datasets generated and/or analysed during the current study are available in the Zenodo repository, https://zenodo.org/record/6342129#.YikDF9XMLIU.

## Abbreviations

PCQ: The Pregnancy and Childbirth Questionnaire
LAS: The Labor Agentry Scale
MADM: The Mothers Autonomy Decision Making Scale
SDM-Q9: The 9-item Shared Decision Making Questionnaire
SDM: Shared decision making
SD: Standard deviation
IQR: Interquartile range
Min: Minimum
Max: Maximum
